# Acute Pancreatitis Secondary to Gestational Hypertriglyceridaemia

**DOI:** 10.1155/2012/627890

**Published:** 2012-07-10

**Authors:** Alexis M. Cahalane, Myles J. Smith, James Ryan, Donal Maguire

**Affiliations:** Department of Hepatobiliary Surgery, St. Vincent's University Hospital, Elm Park, Dublin 4, Ireland

## Abstract

Gestational hypertriglyceridaemia is a rare cause of acute pancreatitis. Its pathophysiology is incompletely understood. Severity scoring and effective management remain challenging. We report a case of acute pancreatitis secondary to gestational hypertriglyceridaemia. We describe the use of computed tomography to provide an alternative determination of severity, as well as plasmapheresis as a means of treating the condition.

## 1. Introduction

Gestational hypertriglyceridaemia (HTG) is a rare cause of acute pancreatitis [1, 2]. The pathophysiology of gestational HTG is attributed to the changing hormonal environment of pregnancy [3], although how this manifests as acute pancreatitis remains to be fully elucidated [4]. A key component in the management approach to acute pancreatitis is the assessment of severity [5]. In this case, given the lipaemic nature of the patient's blood, it was not possible to utilize the more conventional acute pancreatitis scoring schemes. This paper details the use of computed tomography as a means of assessing severity [6], as well as plasmapheresis as a treatment modality to optimise clinical outcome.

## 2. Clinical Case

A 30-year-old, gravida 1 lady, of 39 weeks' gestation, presented to an outside hospital with acute severe epigastric pain, shoulder-tip pain, and severe intravascular volume depletion. An emergency lower section caesarian section was performed, and a healthy female was delivered. During the procedure, the blood was noted to be of “strawberry milkshake” consistency. Blood drawn for analysis precipitated into two layers. The upper two-thirds of the sample, constituting the plasma component, was lipid in nature. The laboratory was initially unable to process the sample, although an amylase of 900 U/L was obtained (reference range: 28–100 U/L). The patient had no significant medical or family history, and her pregnancy was previously uncomplicated. A clinical diagnosis of acute pancreatitis was made based on the revised Atlanta Classification of Acute Pancreatitis [7], most likely secondary to extreme gestational HTG, and the patient was transferred to our institution one day after caesarian section.

Initial examination following transfer revealed a markedly unwell and tachycardic (140BPM) patient with gross abdominal distension and a rigid, tender abdomen. Further investigations demonstrated a leukocytosis (20.5 × 10^9^/L, reference: 3.5–11 × 10^9^/L), elevated random cholesterol (Chol, 20.3 mmol/L, reference: desirable <5.2 mmol/L) and triglyceride (TG, 45.29 mmol/L, reference: 0.15–1.8 mmol/L) levels, and a prothrombin time (PT) of 20 seconds. Lipase (148 U/L, reference: 13–60 U/L), amylase (495 U/L), and C-reactive protein (CRP, 420.0 mg/L, reference: 0–5.0 mg/L) were all elevated. Given the high lipid content of the patient's blood at initial presentation, neither the Ranson or Glasgow scoring systems could be utilised. A contrast-enhanced CT abdomen and pelvis was performed, which found moderate volumes of ascites, small bowel dilatation consistent with ileus, and bilateral pleural effusions with associated atelectasis. There was no radiological evidence of any pancreatic pathology at this stage. As a result, mild acute pancreatitis was predicted as per the Modified CT Severity Index [8].

Despite aggressive resuscitation, the patient remained haemodynamically unstable after transfer (tachycardic and hypotensive), requiring significant vasopressor support. As a result of the associated pulmonary oedema and inotropic dependent cardiac failure, she developed type 1 respiratory failure necessitating intubation and ventilation. In addition, a persistent oliguria secondary to acute renal failure was noted with concomitant metabolic acidosis, and it was felt that dialysis would be appropriate. As a result of this multiorgan failure, the patient was transferred to the intensive care unit. In spite of this, she demonstrated persistent organ failure during her ICU stay [7]. 

Three sessions of plasmapheresis were performed during a progressive deterioration in the patient's condition, using 5% human albumin solution as replacement fluid. She rapidly improved, both clinically and biochemically (random Chol: 3.1 mmol/L, TG: 6.4 mmol/L), was extubated and maintained on supportive care (gastric decompression, jejunal feeding tube, total parenteral nutrition, and haemodialysis), returning to the ward nine days after admission. 

**Figure 1:**
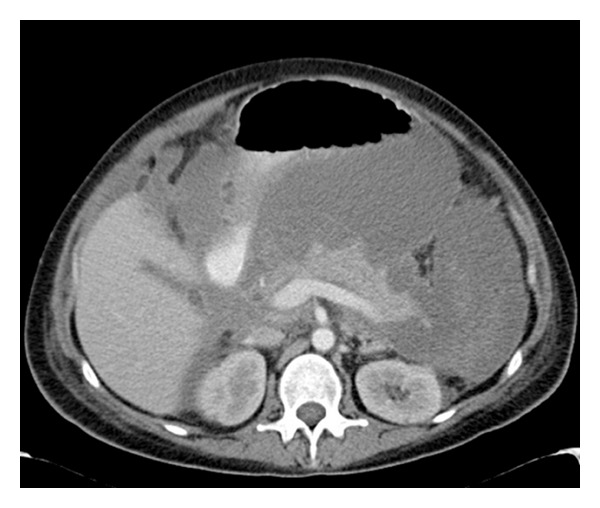
Axial contrast-enhanced CT abdomen 14 days after-admission showing gross ascites and pancreatic parenchymal enhancement.

Once the patient was stabilised, she continued to experience intermittent temperature spikes. Blood cultures were positive for *Staphylococcal* and *Candida* spp. Interval imaging over four weeks showed an evolving ascites (Figure 1) and loculation. No evidence of pancreatic or peripancreatic collections or necrosis was noted during this period. Samples of the ascitic fluid obtained under ultrasound guidance grew *Escherichia coli*. After detailed discussion with the microbiology service, appropriate parenteral antibiotics and antifungals were commenced. The ascitic collections were not amenable to radiological drainage due to their highly loculated nature. A decision was made not to proceed to laparotomy and surgical drainage, as the ascites promptly resolved, and her inflammatory markers quickly fell with the commencement of targeted antimicrobial therapy. The patient's clinical condition continued to improve until the time of her discharge. 

## 3. Discussion

Of particular relevance in this case study is the association between acute pancreatitis and HTG. There is a small, but significant, increased risk of developing acute pancreatitis in individuals with a greatly elevated plasma triglyceride level (greater than 10 mmol/L) [9]. Between 1.3 and 7% of all cases of acute pancreatitis have been attributed to HTG [10, 11], with 1.7–6% of cases of acute pancreatitis in pregnancy attributed to hyperlipidaemia [1, 2]. Interestingly, Syed et al. stated that it is HTG itself that causes acute pancreatitis and not hypercholesterolaemia [12]. 

HTG can be categorized as primary and secondary, although an underlying molecular basis can be found in less than 5% of cases [9]. The secondary forms of hyperlipidemia normally associated with pancreatitis include types I, IV, and V of the Frederickson Classification [13]. Gan et al. also state that 60% of the variability of serum lipid levels is determined by genetic factors [10]. In fact, the typical clinical profile of pancreatitis induced by HTG is someone with a preexisting lipid abnormality in the presence of a precipitant of elevated lipid levels (e.g., poorly controlled diabetes, alcohol use, or a medication) [14]. Pregnancy is a well-known cause of secondary HTG, with cholesterol sometimes increasing by 25–50% and triglycerides by 200–300%, respectively [15]. Ramin et al. found that 53% of all cases of acute pancreatitis in pregnancy, regardless of cause, occurred in the final trimester [16].

**Figure 2:**
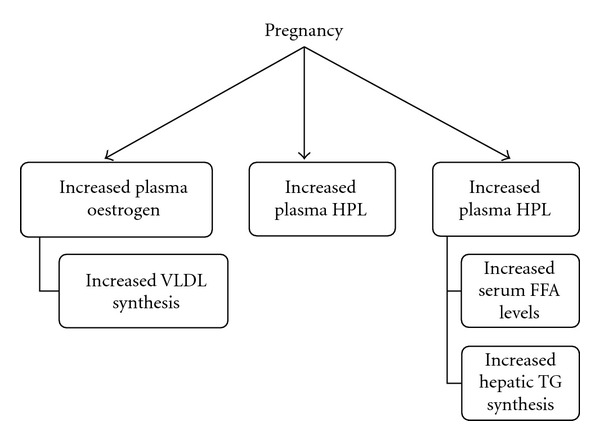
Schematic representation of HTG-related hormonal changes during pregnancy.

The mechanism behind gestational HTG has been widely described. Saravanan et al. suggests that increased levels of serum triglyceride in the third semester of pregnancy can be associated with a similar increase in plasma oestrogen and human placental lactogen (HPL) levels [3]. The increased oestrogen levels lead to increased VLDL production [17], while elevated HPL increases adipose tissue lipolysis and consequently free fatty acid levels in serum [3]. This allows for increased hepatic triglyceride synthesis. Sivakumaran et al. also states that lipoprotein lipase activity is reduced during pregnancy [17]. These factors lead to increased VLDL production and reduced elimination of triglycerides, culminating in elevated serum triglyceride levels (Figure 2). This is exacerbated in individuals with familial HTG, as this additional VLDL production cannot be reversed [3].

**Figure 3:**
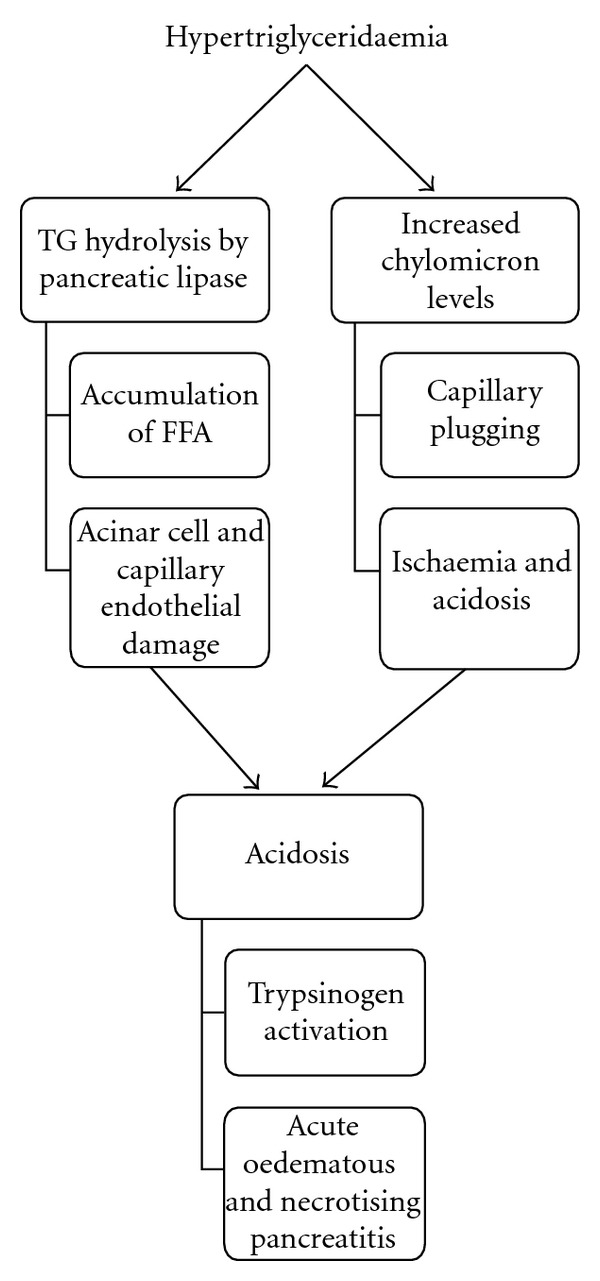
Pathophysiology of acute pancreatitis secondary to HTG.

The exact pathogenesis behind HTG-induced acute pancreatitis is not yet fully understood. Kadikoylu et al. suggested that triglycerides accumulating around the pancreas were hydrolysed by pancreatic lipase seeping out of acinar cells, leading to the accumulation of high levels of free fatty acids [4]. These are believed to be toxic and are thought to damage acinar cells and capillary endothelium. This can occur alongside increased chylomicron concentrations, which can cause capillary plugging, ischaemia, and acidosis. In this acidic environment, free fatty acids activate trypsinogen and trigger acute oedematous and necrotizing pancreatitis (Figure 3). 

Kadikoylu et al. and Bae et al. both state that there is no difference in the clinical course, complications, and physical findings of acute pancreatitis induced by HTG and acute pancreatitis secondary to other factors [4, 18]. However, the latter article does suggest that the HTG element may sometimes contribute to the development of respiratory failure, echoing Warshaw et al. [19].

One essential component in the treatment of acute pancreatitis presentations is the classification of severity at presentation. This is important as, although 80% of presentations are self-limiting mild interstitial pancreatitis that respond satisfactorily to conservative management, a significant minority develop a severe form, with a 10–30% mortality within this cohort [20, 21]. Those patients predicted to develop acute severe pancreatitis normally require high-dependency/intensive-level management, as well as possible intervention. Both clinical and radiological scoring systems have been developed to classify pancreatitis severity [5]. However, in this case, the clinical systems (Ranson's, acute physiology and chronic health evaluation (APACHE)) were unsuitable given the difficulty processing the patient's blood samples on admission. We utilized the radiologically based Modified CT Severity Index [8], which grew out of the initial CT Severity Index described by Balthazar et al. [6, 22]. This scoring system incorporates radiographic features of organ failure and extrapancreatic complications as a means of determining condition severity [8]. However, in this case, the CT was performed within forty-eight hours of onset of symptoms. Warshaw et al. [19] suggested 48–72 hours as an appropriate imaging interval to detect evidence of pancreatic collections and necrosis. While in some cases this may account for the marked inconsistency between the clinical and radiological findings, the clinical team was cognizant of this discrepancy, obtaining interval abdominal imaging to assess the evolution of the condition during the patient's hospitalisation. This emphasized the parallel and complementary nature of clinical and radiological severity scoring and the need to incorporate both into clinical decision making [7]. In fact, this area of acute pancreatitis management remains an evolving field, with continual amendments to the diagnostic criteria and severity classifications being suggested [23].

The initial management of HTG-induced acute pancreatitis is similar to other causes of acute pancreatitis: fasting, intravenous fluids, analgesia, and monitoring for evidence of renal failure or septic shock [4, 18]. Dominguez-Munoz et al. found that serum lipid levels fall in the acute phase of acute pancreatitis, emphasizing the need to check lipid levels early during presentation [24]. Rapidly falling levels of triglycerides may mask the aetiology if investigations are delayed. 

Yadav and Pitchumoni suggest dietary restriction of fats and lipid-lowering agents (preferably fibrates) [14]. However, these methods may take days or weeks before an effect is seen [11]. Gan et al. advocate active steps to lower the levels of precipitating lipid, most notably by removing chylomicrons via plasmapheresis/therapeutic plasma exchange (TPE) [10]. Although first described by Betteridge et al. [25], experience of this method to treat HTG-associated pancreatitis remains limited [14]. The mechanism involves reducing triglyceride levels and circulating activated enzymes, proteases, and inflammatory mediators by physically filtering out these toxic substances from the blood [12]. Chait and Brunzell found that this process could significantly lower plasma lipid levels within two hours [26]. Consistent with earlier studies, Yeh et al. found that roughly two-thirds of cholesterol and triglycerides are removed after a single plasma exchange treatment, with an additional benefit seen in the reduction of triglyceride levels after a second treatment [27]. In this study, 76.5% of hyperlipidemic pancreatitis patients treated with plasma exchange recovered completely. While Yeh et al. did not find that the number of sessions of plasma exchange was linked to survival rates [27], subsequent papers have shown a use for long-term plasma exchange in controlling triglyceride levels [28]. Moreover, the American Society of Apheresis found moderate (category III) evidence supporting plasmapheresis as a treatment of HTG pancreatitis [29]. This was based on a review of several published case reports and series, as well as a randomized controlled trial. Fortson et al. found abscess formation and mortality rates of 13% and 6%, respectively, in their review of HTG-induced acute pancreatitis cases managed supportively [11]. In contrast, Yeh et al. found rates of around 11% in their small case series [27]. This article also found that an increased risk of complications existed when fresh frozen plasma was used as the replacement fluid, compared with human albumin solution. 

In early studies, overall mortality rates for pregnancy complicated by pancreatitis were initially quoted at up to 37% for both mother and child [30]. Foetal mortality rates quoted in the literature have improved in the last twenty years, as earlier studies reflected foetal deaths after preterm delivery which have reduced as a result of improved neonatal care [16]. This study found that 74% of patients suffering acute pancreatitis delivered full-term healthy infants, while a 10.5% foetal mortality rate was noted overall. A more recent study found a perinatal mortality rate of 3.6% [31]. It is, however, important to note that these publications do not distinguish between the various causes of acute pancreatitis during pregnancy. Tang et al. found that those mothers who developed pancreatitis in the first trimester were more likely to experience foetal loss or preterm delivery [32]. A more recent review article found a maternal mortality rate of less than 1% for acute pancreatitis during pregnancy of all aetiologies [31].

Studies comparing the effect of plasmapheresis versus conservative management on morbidity and mortality in cases of HTG-induced acute pancreatitis found no statistical difference [33].

## 4. Conclusion

This paper details HTG of pregnancy as a rare cause of acute pancreatitis. It illustrates the difficulties associated with the diagnosis and prediction of severity in such cases, and the important alternative provided by CT predictive scoring. This specific case is noteworthy due to its rarity and the conflicting data regarding its aetiology and management. We describe the limited role that plasmapheresis can play in the management of similar cases, alongside established medical techniques.
